# Remarkable Case of Osseous Union of the Teeth

**Published:** 1839-06

**Authors:** Eleazar Parmly


### REMARKABLE CASE OF OSSEOUS UNION OF THE TEETH.

By Eleazar Parmly.

A lad about eight years of age, was brought to me by his parents, whose mouth presented the most singular appearance that I had ever witnessed. The incisors were all through the gum, and about one half of the crown of each tooth could be distinctly seen; but they were so irregular in their arrangement, and so remarkable in their form, as to render the mouth so unlike that of other children of the same age, that no person seeing merely an impression or cast of the mouth, would imagine it to belong to a human being. The superior front and lateral incisors were completely and perfectly united both in their enamel and bony structure; the posterior surface showing two distinct teeth, while the anterior presented one smooth and polished surface, excepting at the cutting edge, where there was a slight division. These teeth were as large, and occupied as much space as the four front incisors generally do in the male subject. They were very prominent, and behind them were two other teeth in form and size resembling the lateral incisors.

In order to remedy this ill shapen and unnatural formation and growth, I proposed to the parents to remove the two central incisors, together, of course, with the laterals attached to them, and then by mechanical pressure, to bring the two supernumerary teeth together which stood behind them; and also, bring forward the cuspidati in proper time so as to make a tolerably even arrangement.

I frequently see the young gentleman, whose permanent teeth have now all come in with the exception of the wisdom teeth, and stand in the following order: The two supernumerary teeth occupy the place of the superior front incisors; the cuspidati occupy the position of the lateral incisors; the first bicuspides, the place of the cuspidati, and so on, forming a very pretty arrangement, and one that a casual observer would not notice as differing essentially from other mouths.

I have a two fold object in laying a statement of this case before the profession. The first is to convince parents that in all cases how great soever the irregularity may be, teeth can, by proper care and skill, be brought into systematic order. My second object is to settle a long contested point, whether there does exist any well attested instance of osseous union of the teeth.

Mr. Bell, in his excellent work on the Anatomy of the Teeth, asserts the existence of such union, which is confidently denied by Mr. Koecker. Mr. Bell, in his rejoinder, intimates that Mr. Koecker not only has seen, but may again see, at Guy's Hospital, a perfect example of this kind.—That the specimen which I have in my possession is incontestibly of the same character, no person can doubt, who will take the trouble to inspect it.

I have extracted within the present month, two teeth, the crowns of which were thus completely united.

